# Hypoxic-Ischemic Injury in the Developing Brain: The Role of Reactive Oxygen Species Originating in Mitochondria

**DOI:** 10.1155/2012/542976

**Published:** 2012-03-22

**Authors:** Vadim S. Ten, Anatoly Starkov

**Affiliations:** ^1^Department of Pediatrics, Columbia University, NY, USA; ^2^Department of Neurology and Neuroscience, Cornell University, NY, USA; ^3^Division of Neonatology, Department of Pediatrics, Morgan Stanley Children's Hospital of New York, 3959 Broadway, BHN 1201, New York, NY 10032, USA

## Abstract

Mitochondrial dysfunction is the most fundamental mechanism of cell damage in cerebral hypoxia-ischemia and reperfusion. Mitochondrial respiratory chain (MRC) is increasingly recognized as a source for reactive oxygen species (ROS) in the postischemic tissue. Potentially, ROS originating in MRC can contribute to the reperfusion-driven oxidative stress, promoting mitochondrial membrane permeabilization. The loss of mitochondrial membranes integrity during reperfusion is considered as the major mechanism of secondary energy failure. This paper focuses on current data that support a pathogenic role of ROS originating from mitochondrial respiratory chain in the promotion of secondary energy failure and proposes potential therapeutic strategy against reperfusion-driven oxidative stress following hypoxia-ischemia-reperfusion injury of the developing brain.

## 1. Introduction

Perinatal hypoxic-ischemic (HI) brain injury is one of the most common causes of severe neurological handicap in children. Estimated life-time costs to support children with cerebral palsy, a common outcome of HI brain injury in neonates, reached 11.5 billion dollars in 2003 [1]. Unfortunately, our understanding the mechanisms of the HI brain injury is not deep enough for the development of mechanism-targeted therapeutic interventions in this disease. Even therapeutic mechanisms of post-HI cerebral hypothermia (the only clinically proven neuroprotective strategy) are still not well defined which precludes an optimal use of this potentially powerful strategy. 

**Figure 1:**
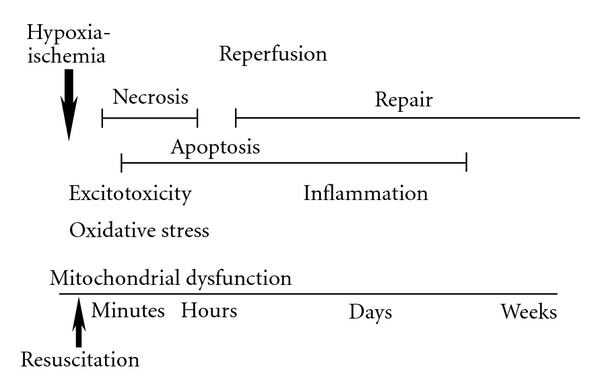
The evolution and major mechanisms of hypoxic-ischemic brain injury. Arrows indicate HI insult and resuscitation (reperfusion), two fundamental events that cause cerebral damage. Different mechanisms may take a lead in the evolution of brain injury: initiated by the bioenergetics mitochondrial dysfunction, cellular injury is driven by excitotoxicity and oxidative stress, followed by the neuroinflammation. The paper is focused on the proximal to the index event mechanism, an oxidative stress and the role of mitochondrial generation of ROS (see text), modified from [2].

Physiologically, HI brain injury could be defined as an acute oxygen and nutrients deprivation to the brain caused by a collapse of cerebral circulation. Hypoxia-ischemia results in severe cellular bioenergetics failure, and if cerebral circulation is not restored, then the brain death is unpreventable. However, if the cerebral circulation is restored for example, as a result of successful resuscitation, then cerebral reperfusion ensures with a full or partial brain recovery. Unfortunately, the same reperfusion can also contribute to the propagation of brain injury initiated by the HI insult. This implies that HI brain injury as a disease, consists of two fundamental pathophysiological events: hypoxia-ischemia and reperfusion. During hypoxia-ischemia and reperfusion mitochondrial dysfunction plays a fundamental role in brain injury. It is now recognized that not only mitochondrial failure to generate ATP during ischemia, but the generation of oxidative radicals and the release of proapoptotic proteins during reperfusion contribute to the cellular damage. The leading molecular mechanisms responsible for the evolution of cell damage and repair during reperfusion change at different timepoints following HI insult (Figure 1). A critical upstream mechanisms to consider in the management of HI brain injury are those linked to an oxidative stress [2]. Therefore, already at the initiation of resuscitation/reperfusion an attempt should be made to limit the reoxygenation-driven burst in generation of reactive oxygen species (ROS) in order to alleviate the severity of oxidative damage to the HI brain. 

## 2. HI and Resuscitation

 It is known that reintroduction of the oxygen to ischemic tissue potentiates oxidative injury. An initial attempt to limit formation of ROS could be made by judicious use of oxygen during resuscitation. Not too long ago, in 2000 the use of 100% oxygen was indisputably recommended for the initiation of resuscitation in all depressed infants [3]. Now neonatologists have tempered their enthusiasm for the use of pure oxygen in neonatal resuscitation. Several clinical trials showed that in the majority of depressed infants the goal of resuscitation, an immediate survival, could be achieved with the use of room air, as effectively as with the use of 100% oxygen [4–6]. Oxygen is indispensable component of ROS. Therefore, regardless of the primary mechanisms of ROS generation during reperfusion, a switch from a routine use of 100% oxygen to the room air at the initiation of neonatal resuscitation, potentially, should limit the severity of an oxidative stress. Indeed, Vento et al. reported a significantly lower level of circulating markers of oxidative stress in neonates resuscitated with the room air (RA) compared to infants resuscitated with the 100% oxygen [7]. However, it remains to be determined to what extent the use of RA in the resuscitation of infants with HI brain injury attenuates an oxidative damage to the brain. Numerous animal studies clearly demonstrated that hyperoxic re-oxygenation maintained for 30–60 minutes of initial reperfusion was detrimental for neurological outcome in asphyxiated pigs and rodents [8–10]. The use of the 100% oxygen in these animals was strongly associated with exacerbation of an oxidative stress in the brain [8]. Of note, however, the hyperoxic resuscitation in these studies was used for 30–60 minutes. At these time-points of reperfusion a full restoration of systemic circulation was already achieved and this resulted in extreme hyperoxemia. Because the primary goal of resuscitation is the return of spontaneous circulation (ROSC), experiments in which the hyperoxic resuscitation is applied beyond the time-point of the ROSC have limited translational importance for the resuscitation science. However, the references cited above do provide an important translational message for the post-resuscitation medical care: All efforts should be made to avoid hyperoxemia in reperfusion. 

Although, normoxic resuscitation has been shown to be effective in the majority of infants, it is still undetermined whether the use of RA in the resuscitation of severely (a complete circulatory arrest) asphyxiated infants is as effective as the use of 100% oxygen in achieving ROSC. After a prolonged (25 minutes) cardiopulmonary arrest in mature pigs, the resuscitation with the use of positive pressure ventilation significantly improved the rate of sustained ROSC and cardiac output only if the resuscitation was supplemented with hyperbaric (~400% O_2_) re-oxygenation [11]. In contrast, following a brief (one minute) cardiac arrest a cardio-pulmonary resuscitation with the use of RA or 100% O_2_ resulted in similar rates of ROSC in neonatal pigs [12, 13]. These data suggest that the duration of circulatory arrest may determine whether positive pressure ventilation needs supplementation with 100% O_2_ to enhance the rate of ROSC. It is critical to understand that no attempts should be made to attenuate a reperfusion-driven oxidative stress at the expense of the efficacy of resuscitation.

Overall, current data suggest that the use of room air in resuscitation reduces the severity of oxidative stress in the majority of depressed infants at risk for HI brain injury. The simplicity of this approach (restriction of oxygen availability for the formation of ROS), however, underscores our incomplete understanding the mechanisms initiating an oxidative injury to the HI brain. Interestingly, Matsiukevich et al. showed that in neonatal mice subjected to a lethal HI insult evidenced by a complete circulatory collapse, hyperoxic resuscitation limited to the time (2 minutes) needed to achieve a sustained ROSC was not associated with exacerbation of reperfusion-driven acceleration in the rate of ROS emission from isolated brain mitochondria [14]. However, it is yet to be clarified whether ROS originating from mitochondria at the onset and during reperfusion cause an oxidative injury to the HI brain. To date, it is still unclear what are sources of pathogenic oxidative radicals in the HI brain, how to enhance antioxidative mechanisms and what are those mechanisms of injury which are initiated or exacerbated by the ROS.

## 3. Potential Sources of Reactive Oxygen Species in HI Injury to the Developing Brain

The evolution of ischemic brain injury following restoration of oxygen and nutrient delivery is a paradoxical biological phenomenon. Although, it is clear that without reperfusion/reoxygenation an ischemic tissue does not survive, maladaptive metabolic changes induced by ischemia predispose cell to dysfunction and death upon reperfusion/reoxygenation. The central role in this phenomenon was assigned to ROS, which can be formed only in the presence of O_2_. Therefore, an oxidative damage occurs mostly upon reintroduction of O_2_ to the ischemic tissue. In the immature brain antioxidant system is underdeveloped which limits inactivation of some ROS and in particular, hydrogen peroxide (reviewed in [2]). The latter is perhaps the most important tissue-damaging ROS species due to its relative stability and the ability to cross lipid membranes. For example, upregulation of Cu/Zn superoxide dismutase (enzyme which converts superoxide into H_2_O_2_) increased, rather than decreased the extent of HI brain injury in neonatal rats [15]. This was associated with elevated level of H_2_O_2_ in the brain. In contrast, transgenic mice overexpressing glutathione peroxidase (enzyme which detoxifies H_2_O_2_ into H_2_O) were markedly protected against HI insult [16]. What is the origin of this H_2_O_2_? What are the major sources of oxidative radicals responsible for an oxidative brain damage in HI? In an elegant study, Abramov and coauthors have identified three distinct ROS generating systems during simulated HI insult (oxygen-glucose deprivation (OGD)) and reperfusion in cultured neurons mitochondrial respiratory chain (MRC), xanthine oxidase and NADPH oxidase [17]. MRC responds to OGD with a burst of ROS emission, which declined by the end of HI insult secondary to a loss of mitochondrial membrane potential. At the end of HI insult a second elevation in cellular ROS generation was attributable to the activity of xanthine oxidase. A third peak in ROS production was due to activity of NADPH oxidase during reperfusion. Inhibition of either NADPH oxidase or xanthine oxidase resulted in a significant neuroprotection [17]. In immature animals and humans with HI brain injury, elevated level of hypoxanthine was proposed as the evidence for a pathogenic role of xanthine oxidase [18, 19]. However, an inhibition of xanthine oxidase with oxypurinol or allopurinol failed to reduce lipid peroxidation, and did not protect the brain in a rat model of HI injury [20] or in human neonates with perinatal HI insult [21]. Genetic or/and pharmacological inhibition of NADPH oxidase also did not exert neuroprotection in different models of perinatal HI brain injury [22]. Taken together these data challenge a pathogenic contribution of NADPH oxidase or xanthine oxidase to an oxidative brain damage following HI in neonates. Interestingly, Loor et al. using a model simulating HI reperfusion injury in cultured cardiomyocytes demonstrated that genetic overexpression of only intramitochondrial ROS-scavenging enzymes, Mn-superoxide dismutase or phospholipid hydroperoxide glutathione peroxidase protected cells against reperfusion-induced death [23]. In contrast, overexpression of Cu-Zn superoxide dismutase or catalase did not result in the protection [23].

Mitochondria are known as a major source for ROS production in the health and diseases, including brain ischemia-reperfusion injury (reviewed in [24]). In mature animal models of ischemia-reperfusion injury to the brain and heart, mitochondria have been increasingly recognized as an important source for the reperfusion-driven acceleration in ROS release [24–27]. However, rapidly emerging evidence supporting a deleterious role of ROS originating in mitochondria during reperfusion are partially counterbalanced by the reports suggesting a prosurvival signaling mediated by mitochondrial ROS in the heart preconditioning ([28], reviewed in [29]) and in postischemic reperfusion [30]. In the developing brain potential deleterious or prosurvival effects of mitochondrial ROS in HI reperfusion were not studied. In the following part of this paper we discuss the experimental data obtained in the mature animal models of the brain and heart ischemia-reperfusion injury which support the primary role of mitochondrial ROS in oxidative damage.

## 4. Mitochondrial ROS and HI Reperfusion Oxidative Stress

**Figure 2:**
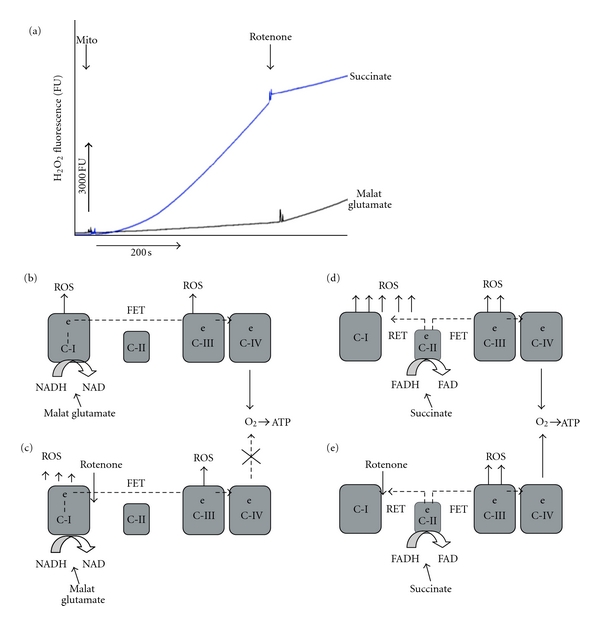
(a) H_2_O_2_ emission rate from brain mitochondria isolated from p10 naïve mouse and supported either with succinate (FAD-linked substrate) or malate-glutamate (NAD-linked substrates). Time-points when mitochondria (mito, 0.05 mg/mL) or rotenone (1 *μ*M) were added are indicated. Cerebral nonsynaptic mitochondria were isolated and mitochondrial H_2_O_2_ fluorescence was measured using Amplex-ultra-red and horse radish peroxidase assay as described in [31]. (b–e), a schematic mechanism for ROS generation in MRC fueled with NAD-linked substrate, before (b) and after inhibition of complex I with rotenone (c), or FAD-linked substrate, before (d) and after rotenone supplementation (e). RET: reverse electron trasport, FET: forward electron transport.

In mature animals several studies detected a reperfusion-driven acceleration in ROS generation from mitochondria associated with oxidative damage to the postischemic heart [25, 26] and brain [27]. A single study showed that in neonatal mice with genetically ablated C1q component of the classical complement activation pathway, the neuroprotection and attenuation of oxidative HI brain injury were associated with the ability of C1q^−/−^ brain mitochondria to release significantly less ROS in response to HI reperfusion, rather then with altered activation of the terminal complement complex [31]. A pathogenic contribution of ROS originating from mitochondria is supported by the data demonstrating that extrinsic or genetic enhancement of mitochondria-targeted ROS scavengers reduces the extent of injury or/and oxidative stress in animal models of ischemia-reperfusion in several organs ([32–34], reviewed in [35]). Furthermore, pharmacological inhibition of ROS generation in the mitochondrial respiratory chain (MRC) limits the extent of ischemia-reperfusion damage and the expression of markers of oxidative injury [26, 36, 37]. These data highlight MRC as a potential target for an antioxidative therapeutic strategy against HI brain injury. In the MRC, complex I and complex III are two major sites for ROS generation during reperfusion [32, 38]. An inhibitory effect of ischemia on complex I has been suggested as a cause for an accelerated generation of ROS in MRC in hearts [26]. However, interpreting the data on postischemic mitochondrial ROS production might be difficult and requires an appropriate experience. The data on mitochondrial function in ischemia-reperfusion mostly were obtained in isolated mitochondria in vitro, when results depended on the choice of experimental conditions. For example, in mitochondria isolated from different organs, including neonatal mouse brain, the response to inhibition of complex I is either increase or dramatic decrease in ROS emission rates, depending upon a substrate used to donate electrons to MRC. NAD-linked substrates such as malate, glutamate, pyruvate, and so forth, invariably support an elevation in mitochondrial ROS emission following an inhibition of complex I with rotenone (Figure 2(a)). In contrast, the use of FAD-linked substrates such as for example, succinate results in robust decrease in mitochondrial ROS emission following an inhibition of complex I with rotenone (Figure 2(a)). These differences in ROS generation by MRC in response to the same complex I inhibitor are well understood and explained by the differences in the electron transport flows, supported by NAD- or FAD-linked substrates (reviewed in [39]). NAD-linked substrates support only forward electron transport flow (FET), from complex I—to membrane-dissolved ubiquinone—to Complex III—to cytochrome c and finally to oxygen through complex IV (cytochrome c oxidase). During this FET, low levels of superoxide can be generated at unspecified MRC sites (likely at complex I and complex III), because some electrons accidentally escape from MRC electron carriers onto O_2_ (Figure 2(b)). Rotenone, pyridaben, thio-barbiturates and other complex I inhibitors interrupt FET between the complex I electron carriers and membrane-dissolved ubiquinone. This interruption of FET increases ROS emission from complex I (Figure 2(c)) secondary to over-reduction of electron carriers (flavin and/or FeS-center N2 and complex I-bound ubiquinone) within this complex (reviewed in [40]). It also stimulates ROS emission from other sources located in the mitochondrial matrix such as for example, dihydrolipoamide dehydrogenase [41, 42], a subcomponent of pyruvate dehydrogenase and ketoglutarate dehydrogenase. This stimulation in ROS production is caused by a decrease in mitochondrial NAD/NADH ratio (as a result of inability of compelx I to oxidize NADH). On the other hand, in the mitochondria fueled with FAD-linked substrates (e.g., succinate) the main electrons flow bypasses Complex I and proceeds from the succinate dehydrogenase (Complex II) to membrane-dissolved ubiquinone, Complex III, cytochrome c, and cytochrome c oxidase. Under specific conditions, such as moderately elevated membrane potential and abundance of FAD-linked substrate, electron flux can—and does—proceed back from complex II, ubiquinone to complex I and further to the matrix-located NAD. This is called reverse electron transport (RET) flow (Figure 2(d)). It was found that RET is associated with very high rates of ROS emission, about 100 folds greater than that obtained with NAD-linked substrates (reviewed in [39]). The major sites of ROS emission in mitochondria fueled with FAD-linked substrate are thought to be complex I and matrix-located enzymes pyruvate dehydrogenase and alpha-ketoglutarate dehydrogenase. Inhibition of complex I with rotenone or similar inhibitors interrupts RET flow and, therefore, substantially diminishes the rate of ROS emission (5–8 folds) (Figures 2(a) and 2(d)) [39]. The RET flow represents the major mechanism for ROS production by mitochondria fueled with succinate, especially in the brain and the heart [43]. It should be noted, that both FET and RET generate proton-motive force and support oxidative phosphorylation of ADP; with RET being about 30% less efficient in terms of energy production but generating tremendously more ROS. 

In vivo, under non-pathological conditions the primary electron donor for MRC in brain mitochondria are NAD-linked substrates for example, pyruvate generated in glycolysis. During ischemia-reperfusion, however, substrate availability significantly differs from that in normal cells. There are several lines of evidence to consider that at the onset of reperfusion postischemic mitochondria actively metabolize succinate. Complex I is the most sensitive among all five complexes to the reduction of the cerebral blood flow, and at the end of ischemia the activity of this complex is significantly reduced [44, 45]. In the immature brain HI resulted in slightly (9% on malate-glutamate) to moderately (21% on pyruvate-malate) greater inhibition of mitochondrial respiration tested on NAD-linked substrates compared to that tested on the FAD-oriented substrate, succinate [46]. In mature rats, forebrain ischemia and six hours of reperfusion resulted in a significant inhibition of mitochondrial respiration tested on NAD-linked substrates. However, no significant differences from the control values were detected when the same mitochondria respired on succinate [47]. This suggests, that after brain ischemia the activity of complex II— is better preserved compared to complex I. This favors a succinate-supported respiration upon reintroduction of O_2_. Indeed, in the rat brain, ischemia resulted in a profound (8–10 fold) depletion of all NAD-linked substrates: pyruvate, citrate, alpha-ketoglutarate, oxaloacetate, fumarate, and malate. In contrast, the concentration of the succinate increased by ~300% [48] and remained elevated at 15 minutes of reperfusion [49]. Following an acute systemic hypoxemia an oxidation of succinate and glutamate by isolated rat brain mitochondria was significantly (>60%) increased [50, 51]. Furthermore, it is known that succinate oxidation inhibits an oxidation of pyruvate and other NAD-linked respiratory substrates, an event associated with over-reduction of mitochondrial pyridine nucleotides [52]. In the heart, the level of succinate also is markedly elevated during ischemia followed by normalization within 30–60 minutes of reperfusion [53, 54], the time-point associated with near-full restoration of mitochondrial metabolic activity in neonatal HI reperfusion [31]. Thus, if at the initial stage of reperfusion mitochondria actively utilize succinate, then interruption of RET flow by complex I inhibiting agents should reduce ROS generation without significant changing ATP-production rate. If the RET flow-dependent production of ROS causes an oxidative damage following HI, then inhibition of complex I recovery upon reperfusion should reduce an oxidative injury. Indeed, in rats with global cerebral ischemia an inhibition of complex I by rotenone or haloperidol significantly reduced tissue accumulation of hydroxyl radicals, resulting in near-complete abrogation of the reperfusion-driven surge in lipid peroxidation products [27]. Ambrosio et al. reported that inhibition of complex I with the thio-barbiturate amytal resulted in significant reduction in the level of free radicals associated with attenuation of lipid peroxidation in isolated rabbit hearts subjected to ischemia-reperfusion [25]. Our data demonstrated that inhibition of complex I with pyridaben significantly reduced cerebral infarct volume and signs of oxidative injury to the brain tissue and mitochondria following HI in neonatal mice [55]. In the model of cardiac arrest and reperfusion, complex I was proposed as a primary generator of ROS [56]. Taken together, these data suggest that ROS generated in complex I participate in oxidative damage to the postischemic brain and heart, making this complex a reasonable therapeutic target against oxidative stress in the early stages of reperfusion. 

In addition to the complex I, complex III has been recognized as an important source for emission of ROS in ischemia and reperfusion [30, 57]. However, experiments with isolated nerve terminals revealed that only very high level of complex III inhibition (70–80%) resulted in detectable elevation in generation of H_2_O_2_ [58]. Given, that after brain ischemia mitochondrial respiration on succinate was shown to be markedly better preserved compared to that tested on complex I linked substrates [47], the rationale to consider complex III as a therapeutic target in reperfusion is weak. Indeed, in mitochondria respiring on succinate the RET flow (complex I) contribute the most to ROS production. Finally, it is unrealistic to inhibit complex III without robust reduction in production of ATP which could be detrimental for the tissue recovery. 

## 5. The Pathogenic Mechanisms Targeted by Mitochondrial ROS in HI Reperfusion

Traditionally, a detrimental effect of oxidative stress is supported by evidence of structural oxidative alterations to the post-HI brain. However, it is also important to determine what specific mechanism of injury could be targeted by ROS during reperfusion. In the design of neuroprotective strategies, it is not only a source of injurious ROS, but also a particular mechanism of damage triggered/exacerbated by these ROS is important to consider. Logistically, if an oxidative stress is one of the earliest reperfusion-driven damaging events, the mechanism targeted by ROS should be in close temporal proximity to the index event.

 In the ischemic brain, cells experience glutamate-receptors over-stimulation and cellular Ca^++^ overload, which occurs to a markedly greater extent in the neonatal brain than in the mature CNS [59, 60]. Mitochondria actively participate in preservation of cellular Ca^++^ homeostasis by up take of Ca^++^ from the cytosol into mitochondrial matrix space (reviewed in [61]). However, if mitochondrial Ca^++^ load exceeds mitochondrial capacity to hold Ca^++^, then mitochondrial membranes loose their integrity via opening a channel in the inner membrane, termed the mitochondrial permeability transition pore (mPTP). Transient and permanent opening of mPTP has been strongly considered as one of the leading mechanisms of necrotic and apoptotic cell death in the brain and other organs following ischemia-reperfusion injury ([62, 63], reviewed in [64]). It has been shown, that mitochondrial ROS can initiate an opening of mPTP during ischemia [22] and reperfusion [65, 66] even in the absence of cyclophilin-D (the only known structural component of mPTP) or Ca^++^ overload [67, 68]. Mitochondria-targeted antioxidant, mitoTEMPO, partially prevented mPTP opening and attenuated necrosis and apoptosis following simulated ischemia-reperfusion injury in cultured renal tubular cells [69]. Taken together these data suggest, that regardless of the type of the organ, ROS originating from mitochondria upon reperfusion can trigger a loss of integrity in mitochondrial inner membrane, the event suggested as the "point of no return" in propagation of cell death following HI insult.

## 6. The Role of Mitochondrial Membrane Permeabilization in the HI Brain Injury

### 6.1. Inner Mitochondrial Membrane Permeability Transition Pore (mPTP) and HI Injury in the Developing Brain

 Independent of the developmental stage, HI insult severely inhibits mitochondrial oxidative phosphorylation. It has been shown that in immature brain, at the end of HI insult mitochondrial phosphorylating respiration was significantly suppressed [31, 70, 71]. Reoxygenation/reperfusion restores mitochondrial ADP-phosphorylating capacity, normalizing ATP content in the post-HI brain. However, following several hours of reperfusion mitochondria exhibit a profound decline in their ADP-phosphorylating respiration rates [31, 46], the event known as a secondary energy failure. The molecular mechanism proposed to explain the pathogenesis of secondary energy failure is opening of mPTP. mPTP renders organelles incapable of ATP production due to a loss of proton-motive force and NAD. This bioenergetics failure results in mitochondrial swelling, leading to a permeabilization of the outer mitochondrial membrane and release of pro-apoptotic proteins which eventuates in necrotic and apoptotic cell death [72–74]. It has been shown that in neonatal rats inner mitochondrial membrane opens mPTP at 0–1.5 hours and at 6–8 hours after HI [75]. However, the pathogenic significance of mPTP in the reperfusion injury in the developing HI brain remains uncertain. For example, as opposite to adult mice, neonatal cyclophilin-D knock-out mice were found to be susceptible to HI injury [76]. Earlier the same group has reported that antagonist of cyclophilin-D, cyclosporin-A did not attenuate the extent of HI brain damage in neonatal rats [77]. In contrast, using the same model Hwang et al. reported that cyclosporin-A, injected immediately after HI insult significantly protected developing brain, attenuating both necrotic and apoptotic cell death in neonatal rats [78]. Similar results were obtained in neonatal rats subjected to a mild focal cerebral ischemia-reperfusion [79]. In neonatal rats and mice subjected to a global hypoxia-ischemia-reperfusion injury, a post-treatment with cyclosporine A markedly potentiated the neuroprotective effect of Ca^++^ channel antagonist, nimodipine [80]. Given, that in mature animal models of ischemia-reperfusion injury a pathogenic role for mPTP has been strongly suggested, more extensive research is needed to clarify the contribution of mPTP opening to cerebral HI reperfusion injury in the developing brain.

### 6.2. Outer Mitochondrial Membrane Pore (OMMP) and HI Injury to the Developing Brain

 Following an ischemic insult mitochondrial membrane permeabilization can occur via opening of outer mitochondrial membrane pore (OMMP) induced by Bak/Bax translocation into mitochondria. This pore is thought to be primarily responsible for a release of pro-apoptotic proteins from the mitochondrial inter-membrane space, leading to an apoptotic cell death [81, 82], including that induced by an oxidative stress ([83], reviewed in [84]). Importantly, in HI reperfusion injury to the developing brain Bax dependent OMMP has been suggested as a primary mechanism of injury ([76], reviewed in [85]). Developmental shift toward a priority of the Bax-dependent OMMP over the cyclophylin-D dependent mPTP opening in the HI brain damage has been supported by the data obtained in cyclophilin D knock-out neonatal mice [76], as well as by neuroprotective effect of Bax-inhibiting peptide [86]. However, in contrast to a better understanding of events leading to secondary energy failure and necrotic cell death following an opening of mPTP, it is less clear how Bax/Bak mediated OMMP opening affects oxidative phosphorylation and results in secondary energy failure and necrosis. One possibility is that postischemic opening of OMMP results in a massive loss of cytochrome c from the inter-membrane mitochondrial space which results in secondary inhibition of oxidative phosphorylation. However, this loss of cytochrome c was not mediated by mPTP opening, and was not associated with changes in mitochondrial Bax, Bad, Bak or Bid [87]. Although, mitochondrial ROS appeared to be critical for the execution of Bax/Bak dependent apoptosis induced by anti-cancer drugs [88, 89], we have not found data that ROS originating in mitochondria are involved in the Bax/Bak-induced apoptosis in HI brain injury. Interestingly, oxidative stress-induced cell apoptosis clearly required the presence of ROS originating from MRC to signal mPTP opening, but this apoptosis was independent of Bax translocation [90]. The existence of two relatively independent mechanisms of mitochondrial membrane permeabilization does not exclude the contribution of each of these mechanisms in HI damage to the developing brain. Indeed, there is evidence for involvement of cyclophilin D dependent mPTP opening in the Bax-driven cytochrome c release in the isolated mitochondria [91].

**Figure 3:**
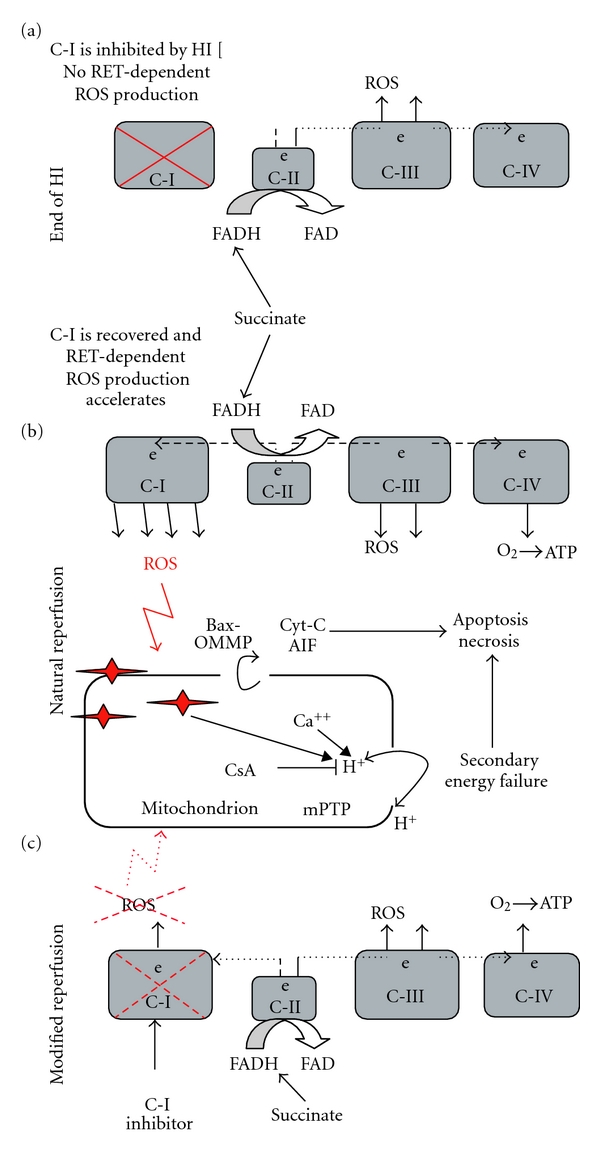
Proposed mechanisms of HI injury targeted by mitochondrial ROS and mechanisms of ROS generation in the MRC at the end of HI insult (a), at the initiation of a natural reperfusion (b), and the reperfusion therapeutically modified by an inhibition of the reperfusion-driven complex I recovery (c). Arrows indicate the leak of cytochrome c and apoptosis inducing factor (AIF), the loss of proton motive force and Ca^++^ and ROS contribution to the mPTP opening. CsA is a cyclosporine A which partially inhibits mPTP.

In conclusion, the analysis of current data supports the hypothesis that in the developing HI brain reoxygenation/reperfusion causes not only recovery of cell bioenergetics, but also accelerates ROS generation in mitochondrial respiratory chain (Figures 3(a) and 3(b)). These ROS can cause an oxidative damage to mitochondrial membranes. This damage occurs in the forms of mPTP and Bax/Bak dependent outer membrane pores, both of which are considered as a “point of no return” in the evolution of HI injury. With data that complex I contributes to accelerated generation of ROS during reperfusion, a novel neuroprotective strategy against reperfusion-driven mitochondrial membrane permeabilization may consist of reversible pharmacological inhibition of complex I recovery following HI insult (Figure 3(c)).
